# The significance of a dialectical approach to enrich health professions education

**DOI:** 10.1186/s12909-024-06108-4

**Published:** 2024-10-07

**Authors:** M. C. de Ruiter, L.-M. van Klaveren, V. G. M. Geukers

**Affiliations:** 1 Faculty of Medicine, Institute for Education and Training, Amsterdam UMC, University of Amsterdam, Meibergdreef 9, 1105 AZ Amsterdam, The Netherlands; 2 Amsterdam UMC, Emma Children’s Hospital, University of Amsterdam, Meibergdreef 9, 1105 AZ Amsterdam, The Netherlands

**Keywords:** Professional competence, Health professions education, Interprofessional education, Dialogue, Dialectics, Instructional design

## Abstract

The Lancet Global Independent Commission has called for a systems-based approach to health professions education. They emphasised the acquisition of collaborative skills, critical reasoning and ethical conduct to prepare students for interprofessional collaborative practice (IPCP). Interprofessional education (IPE) has been put forward as a promising strategy. However, despite the global efforts to incorporate IPE in health professions education curricula, the evidence for a positive impact on IPCP is still inconclusive. This may be related to the misalignment between competency-driven IPE programs that focus on end-stage professional competence and the non-linear development of students’ competence that is necessary for the dynamical nature of IPCP. Therefore, we argue that health professions education, and IPE in particular, needs to incorporate these dynamical processes including social and organization sensitivity. We present a conceptual framework that integrates the Cultural-Historical Theory, the principles of dialectical thinking and the concept of metastable attunement. While dialectical thinking is the ability to perceive the complexity of a dynamic reality that is in a state of constant transition, metastable attunement refers to the consequent adjustment to it. The subsequent instructional design employs a dialectical approach to teaching and learning, based on mediating activities and dialectical inquiry. To reach the full potential of this approach, the mediating activities should ensure a continuum of learning across the curriculum. In addition, faculty development needs to focus on the principles of dialectical inquiry as a pedagogy to optimally guide students. Further research into the extent to which healthcare professionals and students think dialectically may inform improvements to the proposed instructional design, the structure of the learning continuum and the essential requirements for faculty development.

## Introduction

Over the past few decades, the World Health Organization (WHO) has advocated to strengthen healthcare systems in order to address global health needs more effectively [1]. Interprofessional collaborative practice (IPCP) presents a promising strategy to reach these goals [2, 3]. It is based on the assumption that healthcare professionals with common values, attitudes and behaviours are able to provide optimal care to sustain health and well-being of all people [3]. To prepare students for IPCP, the Lancet Global Independent Commission [3] has proposed a systems-based approach to health professions education, with a greater emphasis on collaborative skills, critical reasoning, and ethical conduct alongside competency-driven approaches to instructional design [4]. In this context, interprofessional education (IPE) has been identified as a potential educational strategy to develop skills, attitudes and adaptive capacity that are essential for ICPC [2–4]. During IPE, students from two or more professions learn with, from and about each other [2, 5].

To date, IPE has been incorporated into a multitude of health professions education curricula and healthcare settings across the globe [6, 7]. In recent years, research has increasingly focussed on instructional design with a particular emphasis on the definition of learning outcomes [8], the understanding of learning in interprofessional teams [9], and the identification of mechanisms that explain students’ changes in behaviour [10, 11]. However, despite these considerable global efforts and the positive effects that have been observed, the evidence that IPE improves IPCP remains inconclusive, especially at the higher Kirkpatrick levels such as skill development and behavioural transformation [12–14]. The number of studies that measure patient outcomes is limited, with a heterogeneity in methodologies and outcome measures [13]. Moreover, the effect of practice-based interventions is uncertain, and learning processes are still under-studied [14].

Given the complex and dynamic nature of IPCP and the non-linear development of students’ competence [10, 15, 16], we argue that a competency-driven approach to IPE alone is insufficient to adequately prepare students for ICPC. While competency frameworks (e.g., CanMEDS Framework [17]; Canadian Interprofessional Health Collaborative (CIHC) Framework [18]) describe the end-stage competence of qualified healthcare professionals, they do not explicate the long-term, iterative learning processes [15, 19]. However, competency development is a continuous cognitive and social process that involves perception and (inter)action at multiple levels [20]. It is therefore essential to develop both professional expertise and social skills in order to competently navigate the nuances of clinical practice [21] and to collaborate effectively across professional boundaries in unpredictable and uncertain settings [3, 16, 19]. Consequently, learning to participate in the complex and dynamic healthcare system requires a long-term and relational approach [16, 19]. This approach enables the constant transformation within the (inter)professional discourse [3, 22] and the development of social and organisational sensitivity [21].

In order to facilitate the processes of (inter)professional collaborative learning, we put forward a coherent conceptual framework to the instructional design of health professions education, including IPE. This framework integrates two theoretical approaches: First, the Activity Theory elucidates the dynamics of shared practice such as IPCP and IPE [23–25]. Second, the principles of dialectical thinking describe the ability to perceive the complexity inherent in IPCP and IPE [25]. In addition, we use the concept of metastable attunement to further outline this ability as a constant and effective adjustment to particular situations [4, 26, 27]. With the conceptual outline of the dialectical approach, we aim to enrich health professions education, and to align IPE to IPCP more effectively.

## Activity theory

The Activity Theory offers insights into learning processes and interrelationships inherent to a dynamic shared practice [6, 28]. The theory has two distinct traditions, being Cultural-Historical Activity Theory [29] and Activity Hierarchy [30]. Both are based on the Cultural-Historical Theory of Vygotsky [24, 31], which posits that the development of higher mental functions, including self-regulatory behaviour, is influenced by and dependent on the dynamics of social interaction [25, 31].

**Fig. 1:**
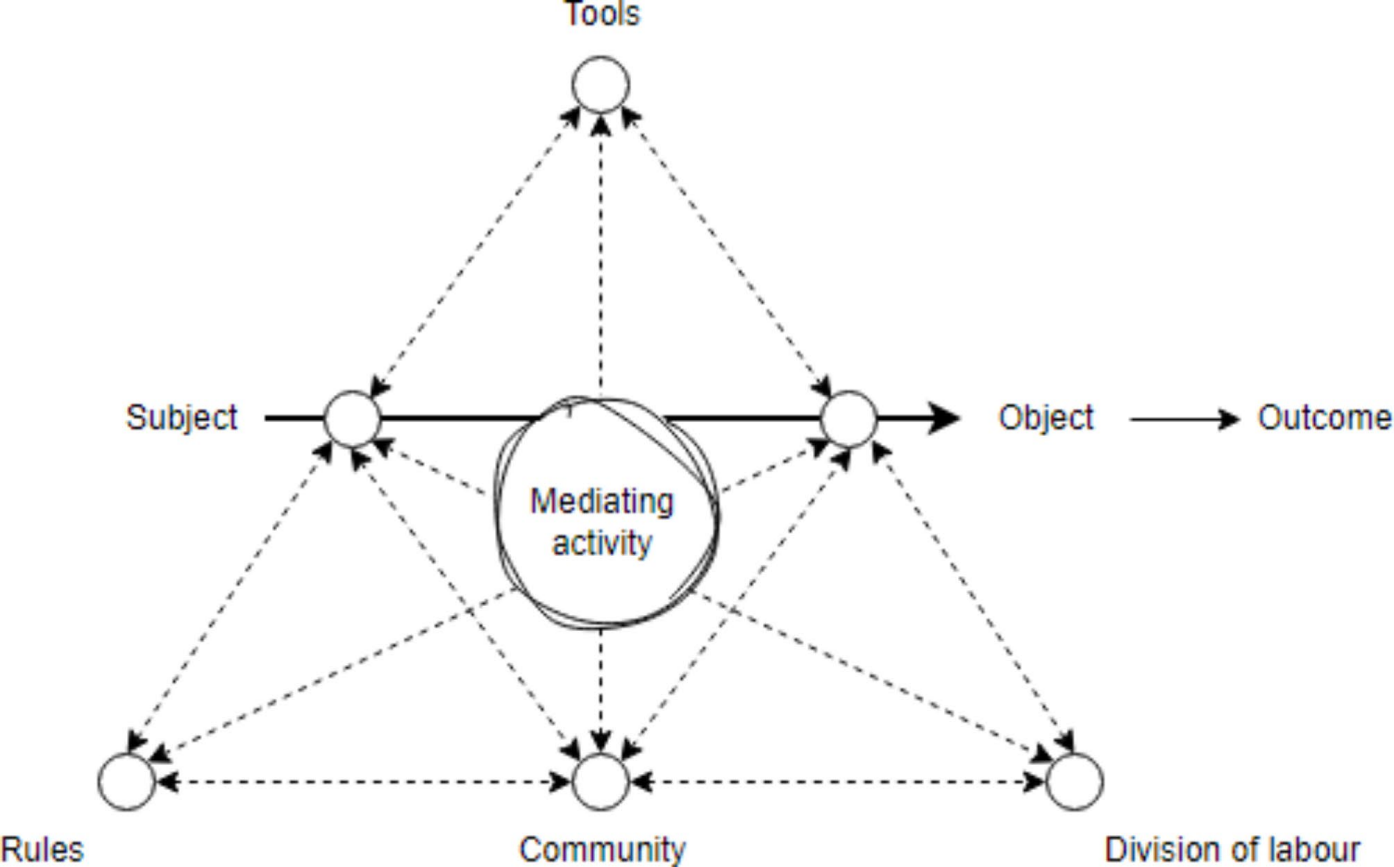
The adapted meta-theoretical framework of Cultural-Historical Activity Theory. *Adapted from Cong-Lem*, *N. Vygotsky’s*, *Leontiev’s and Engeström’s Cultural-Historical (Activity) Theories: Overview*, *Clarifications and Implications. Integrative psychological & behavioral science. 2022;56*, *1091–1112* [24].

The Cultural-Historical Activity Theory [29] is a meta-theoretical framework that is frequently employed for instructional design and research [32]. The framework is composed of seven interrelated elements: subject, object, tools, community, rules, division of labour, and outcome (see Fig. 1) [23, 24]. It explains the dynamic interplay between individual subjects engaged in shared practice within a socio-cultural environment, which is defined as community [29]. Each individual subject within this community focuses on the objective of the shared practice. To regulate their activities, and to achieve the objective, the interacting individuals divide labour, adopt common rules, and use tools for interaction [29, 31]. These tools may include language or a timeline for planning their actions [31]. The outcome of the shared practice is based on the intended or unintended consequence(s) of the dynamic interplay.

**Fig. 2:**
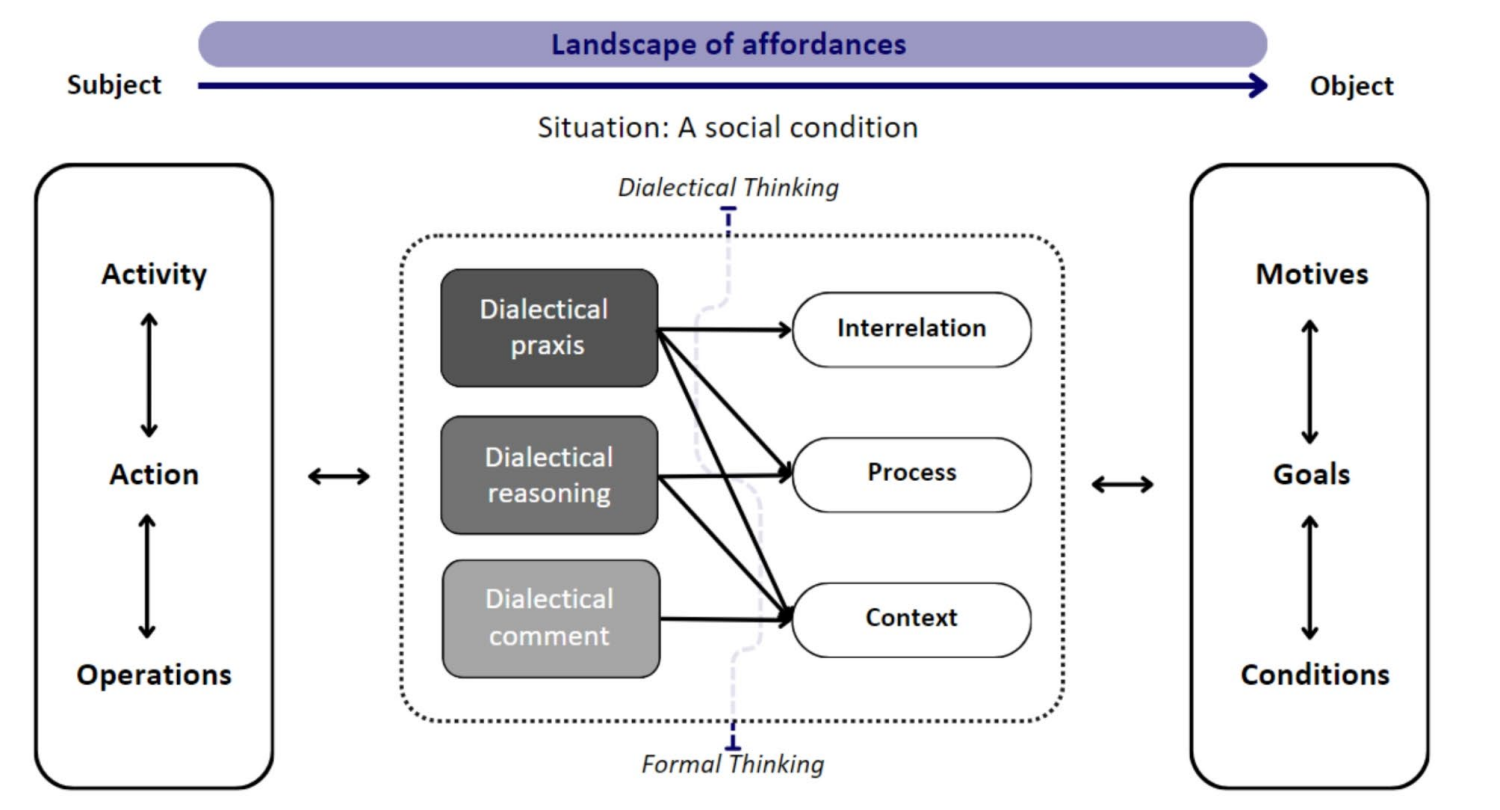
The integrated model of dialectical interrelations associated with an activity. *Adapted from Hasan*, *H. & Kazlauskas*, *A. Activity Theory: who is doing what*, *why and how. In H. Hasan (Eds.)*, *Being Practical with Theory: A Window into Business Research. 2014. (pp. 9–14). Wollongong*, *Australia: THEORIE.* [23]

The Activity Hierarchy [30] understands shared practice as intentional and goal-oriented actions to bring about change [25, 28, 29]. Leontiev [30] argues that individuals involved in an activity are dialectically interrelated, driven by their own motives, goals and conditions (see Fig. 2) [23, 25, 28]. Motives refer to the underlying reasons for engaging in an activity, while conditions relate to mental and physical operations that are necessary to achieve a particular goal through certain actions [30]. Actions of individual subjects may diverge and/or converge during the process of working towards the shared practice‘s objective [28, 33]. A possible positive outcome is a shift in focus from individual actions to more reciprocal and meaningful forms of participation [28, 33]. In addition, the objective may undergo a transformation as a result of the collective response to the dilemmas or tensions based on perceived socio-cultural contradictions [24, 28, 29, 33].

In this respect, the concept of boundary-crossing highlights the significance of acknowledging dilemmas or tensions in social interaction and overcoming contradictions in thoughts, beliefs and values [28, 34]. It describes four dialogical learning mechanisms that may be triggered when individuals engage in boundary-crossing activities such as the collaboration with unfamiliar professions. These mechanisms include identification, coordination, reflection, and transformation [34, 35]. Through meaningful interactions in boundary-crossing activities, individuals may develop the adaptive capacity to function competently in various shared practices [34, 36]. Therefore, this process of learning may also be considered a transformative praxis that facilitates dealing with perceived socio-cultural contradictions [34, 37].

In the context of IPE, activities that mediate interactive dialogue between students from different professions with the objective to achieve a common goal may facilitate cognitive and social development [28, 31, 35]. Figure 1 illustrates a mediating activity as the dynamic interplay within a shared practice [31]. The dashed arrows illustrate the dialectical interrelations that facilitate interactive dialogue. The continuous arrow represents the focus of the individual subject towards the objective of the activity. Examples of such mediating activities include collaborative clinical reasoning to create an integrated care plan [38] and collectively mapping a patient journey [39].

Importantly, both Activity Theory and the concept of boundary-crossing underline that shared practices are inherently dynamic processes with uncertain outcomes [29, 34]. Therefore, the realisation of objectives in shared practices relies on the awareness of students‘ interdependence and adaptability [24, 25] (see Figs. 1 and 2). In particular, the dialectical interrelations in the interprofessional dialogic space [35] can be considered a ‘zone of complexity’ [6]. In order to develop new perspectives and to understand the value of shared knowledge, dialogic agreement and mutual enrichment, active participation in discourse is imperative [40–42]. This engagement involves critical-dialectical discourse on boundaries in practice [40, 43] and conscious self-reflection on the reciprocal relationships between the context, the self and the others, taking into account the unique habits of mind and socio-cultural experiences [43, 44]. In turn, this may facilitate two essential processes for IPCP: First, the formation of a common view on the shared practice at hand and its anticipated outcomes. Second, the capacity for constant adjustment to a dynamic environment [3, 4].

In conclusion, following the Activity Theory, IPE is a complex shared practice as a result of the socio-cultural environment and the motives of individual students. In order to achieve the objective of the activity, students must be aware of the need to actively explore their interdependence, resolve contradictions in their thoughts, beliefs, and values, and be willing and able to adapt. Finally, active engagement in critical-dialectical discourse and conscious self-reflection may facilitate students’ transformative praxis.

## Dialectical thinking

Dialectic is a philosophical concept based on the principles of dialogue [42, 45]. It assumes that reasoning serves as a means to comprehend the dynamic reality as an integrated whole of interrelated elements in a continuous process of emergent change that is characterised by both differentiation and integration [25, 46]. The principles of dialectical thinking describe the ability to perceive the complexity inherent in IPCP and IPE.

While formal logical-analytical thinking aims to exclude contradictions from a closed-system perspective, dialectical thinking stays open to the logic of opposites that are present in the complex and dynamic reality [46–49]. It refers to the ‘ability to perceive things as developing and changing, to understand the causes of their change, and to perceive the trends and directions of their future evolution’ [50], and views phenomena from a universal developmental perspective [42, 46]. The awareness of what is yet to come — the consciousness of absence — plays a key role in dialectical thinking [37, 48].

**Fig. 3:**
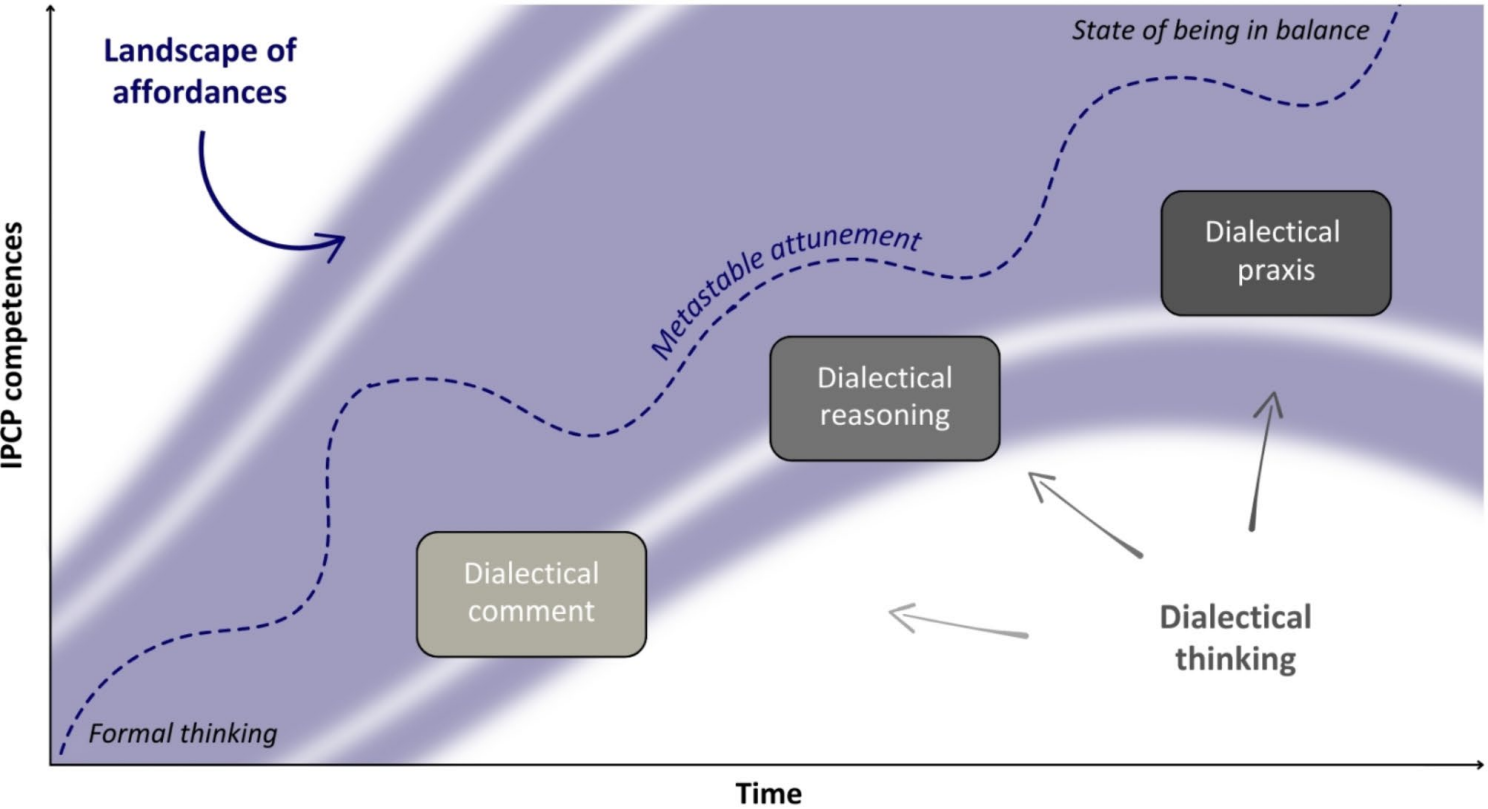
The development of dialectical thinking

Bhaskar [37] argues that the development of dialectical thinking builds on formal logical-analytical thinking and follows an accumulative series of stages: dialectical comment, dialectical reasoning and dialectical praxis (see Fig. 2). Learning through these stages of dialectical thinking is a non-linear process that occurs via multiple iterations due to repeated practice and the revision of thoughts. In the first stage, students develop the awareness of the limitations of one’s own thought [37]. Such dialectical comments focus on the specific context and view opposites as discrete entities that exist independently of spatial, temporal, and transformative constraints [37]. Building upon dialectical comments, students’ frames of reference may start to shift [37] [48]. This stage of dialectical reasoning involves a progressive deepening of one’s comprehension of reality as a process of change, which is influenced by the lived experiences and the situation at hand [37, 51]. By acknowledging and constantly questioning assumptions, values, motives and actions, dialectical reasoning may lead to a more profound perspective on reality and the identification of interrelations [37, 48, 52]. In the final stage, individuals engage in the iterative process of adjustment to the independent contexts, processes of change and interrelations, known as dialectical praxis (see Figs. 2 and 3) [53]. Bhaskar [37] describes dialectical praxis as an automatic reciprocal process of reflection on the perceived reality as an integrated whole in a state of constant transition, including its potential prospects. It is an open, active and reflexive approach and has transformative nature [37]. The synthesis of formal logical-analytical and dialectical thinking may consequently result in a more inclusive perspective on shared practice [37]. In its ultimate form, the perception of opposites is no longer evident, resulting in a state of being that is in balance with the dynamic reality [37, 54].

Figure 2 illustrates the link between activity, actions and operations (left box) with motives, goals and conditions (right box) proposed by Activity Hierarchy [30] via the capacity for dialectical thinking (central box). This capacity builds on formal logical-analytical thinking [37, 48], which is exemplified as formal thinking. Dialectical thinking is specified by comment in relation to context, reasoning in relation to change, and praxis in relation to interrelations [37]. At the levels of dialectical comment and reasoning, the reflection encompasses aspects of reality. In contrast, dialectical praxis is transformative, based on reflection on the activity as a whole [37]. Moreover, the motive to engage in the activity is influenced by a social condition [30].

Dialectical praxis may be conceptualised as a form of metastable attunement, which enables the capacity for constant adjustment to unpredictable and uncertain situations in complex dynamic environments [26, 53]. Metastable attunement describes the state of being and staying in balance as two iterative steps [26, 27]: First, the perception of a wide range of possibilities for action in a specific environment in relation to the perceivers‘ capacity — landscapes of affordances— (see Figs. 2 and 3). Second, the consequent selection of the most appropriate affordances in a given situation to ensure effective and efficient outcomes over time. Based on their ability to discern the subtleties in a particular situation, dialectical thinkers may perceive a wide range of action possibilities that enables them to navigate a complex and dynamic environment flexibly and creatively, and to explore novel approaches for action [27]. Research shows that dialectical thinkers adapt to new environments more effectively and employ a range of coping strategies in complex situations [55, 56]. They consider the dynamic context and interrelations during shared practices, which has a positive impact on their performance [57]. Furthermore, dialectical thinkers are aware that their verbal and non-verbal actions may affect others, as well as impact their future relationships with them [37, 45]. However, not all individuals reach the final stage of dialectical thinking and consequent metastable attunement [46, 47]. As a result, a significant number of students and healthcare professionals may not be aware of the limitations of their own thinking and the potential impact on practice [51, 58], which is relevant to IPCP [54].

Nevertheless, previous literature indicates that the development of dialectical thinking and its constant employment as metastable attunement can be cultivated [26, 59, 60] and may enhance students’ intrinsic motivation to learn and practise [56, 61].

## Dialectical approach as instructional design

To strengthen the collaborative competence of (future) healthcare professionals and to align IPE with IPCP more effectively, we argue for the adoption of the dialectical approach as a coherent conceptual framework for the instructional design alongside competency-driven approaches in health professions education. This framework explicates the non-linear development of dialectical thinking in a complex and dynamic environment over time (see Fig. 3) and the consequent need for guidance. The curved dashed arrow in Fig. 3 illustrates the progression of dialectical thinking and metastable attunement. This process begins at the level of formal logical-analytical thinking, which is exemplified by formal thinking. Dialectical thinking is exemplified by the levels of dialectical comment, dialectical reasoning and dialectical praxis [37]. The ultimate form of dialectical praxis is a state of being in balance with reality [37].

Key to the dialectical approach to teaching and learning is the use of mediating activities that allow for dialectical inquiry into the context, processes of change and interrelations of the situation at hand [27, 62–64]. Effective mediating activities enable students to actively explore a range of affordances and practise to select of the most appropriate action in a given situation [20], and provide students with situations that require receptivity and intentional actions [27]. These activities must ensure an objective that addresses interactive dialogue, allowing for the exchange of opinions, assumptions, and experiences [61, 63, 65], and become increasingly more complex in nature over time. A dialectical task, which challenges students to deal with potential opposites in thought and practice, is an example of such an objective [52, 66].

The aim is to facilitate dialectical transformations of thought in terms of context, processes of change and interrelation (see Fig. 2) [43, 44]. The dynamic interplay at the core of this process of learning is the awareness of one’s own perceptions and thoughts [65] through a dialectical process of self-reflection that addresses the functioning of the shared practice (see Figs. 1 and 2). At a deeper more profound level, the self-reflection may include the perceived opposites in thoughts, motives and values pertaining to the self and others [34], as well as the perceived affordances within the particular situation [26, 27]. This progression in dialectical thinking is exemplified through the manifestation of dialectical comments, dialectical reasoning and, ultimately, dialectical praxis.

The integration of a dialectical process of inquiry within a wide range of dynamic situations [64, 67] across the curriculum, both preclinical and clinical, such as (inter)disciplinary, (inter)professional and international learning environments and internships, may foster the accumulative development of dialectical thinking. Therefore, this dialectical approach to teaching and learning should be implemented at an early stage (see Fig. 3). In addition, healthcare professionals and educators must be adequately prepared for dialectical inquiry aimed at context, processes of change and interrelation, and the guidance of students through changing landscapes of affordances and intentional actions (see Figs. 2 and 3). To optimise the (inter)professional learning experience, it is essential that they encourage the autonomy of students [12] and foster dialectical inquiry and dialectical self-reflection [65, 68, 69]. By engaging in sustained dialogue that encompasses the context, the processes of change, and the interrelations in shared practice, healthcare professionals and educators could foster a shift in perspective towards a more inclusive mode of reasoning on a reality in constant transition [37]. Potential pedagogical approaches include the use of dialogical learning techniques, such as Exploratory Talk and Accountable Talk [41, 70].

## Discussion

In this paper, we put forward a coherent conceptual framework to enrich health professions education. The framework is based on the Cultural-Historical Theory [31], the principles of dialectical thinking [37] and the concept of metastable attunement [26, 27]. The aim is to foster the development of IPCP competence through dialectical transformations of thought in terms of context, processes of change and interrelation, and thus enabling students to attune to a complex and dynamic healthcare system.

By taking the mediating activity and dialectical inquiry as starting point, we have expanded the Activity Theory with dialectical thinking. The consequent instructional design integrates the Structural Dialectical Approach to Cognition [52, 72] and the Dialectical Approach to Inquiry [46, 52, 71], but excludes the naive approach to dialectical thinking, which is culturally based and inclined to accept perceived contradictions [52, 59, 73]. In line with Veraksa [52], we assume the dialectical logic of objects as a transformable unity of opposites that can coexist [49, 52]. Empirical research suggests that the development of dialectical thinking follows Bhaskar’s dialectic in a fluid way [48, 71]. This is pertinent to our conceptual framework as illustrated in Figs. 2 and 3. Furthermore, by incorporating complexity and values, and with an understanding of absence as a key element, the dialectical approach provides a perspective on professional development that has the potential to facilitate transformative change in practice and better align IPE to IPCP [37, 54].

Within the dialectical approach, interactive dialogue and dialectical reflection are indispensable to the process of deriving meaning from a dynamic experience [43, 44]. While the dialectical pedagogy of ‘teaching-learning’ draws on both didactic approaches [74], it may not yet be widely known among healthcare professionals and educators. This may have implications for the integration of the new dialectical approach into the curricula of health professions education. Furthermore, the design of mediating activities for active exploration in accordance with the principles of dialectical thinking [37] and the concept of metastable attunement [26, 27], may present a challenge for healthcare professionals and educators that are unfamiliar to it.

At the moment it is unclear to what extent healthcare professionals perceive situations as complex and unpredictable, and whether healthcare professionals and educators apply dialectical thinking. The latter could be quantified through the use of instruments designed to assess the level of dialectical thinking, such as the Dialectical Thought Form Framework [48] and the Dialectical Self Scale [75]. However, it should be noted that the choice of instruments must be aligned with the situation under study. For example, the Dialectical Self Scale is based on the naïve dialectical approach [73], and therefore may not be applicable in all settings.

Despite the indications of its potential, at present, there is no evidence to prove that the proposed dialectical approach to teaching and learning will be an effective instructional design for facilitating change in behaviour or for aligning health professions education to IPCP. However, faculty development seems important, particularly given the fact that developing a sensitivity to dynamic social interactions through dialectical thinking is a long-term, relational process of learning [31, 47, 48, 51] that depends on the socio-cultural and learning environment [66, 72, 76, 77].

## Conclusion

In this paper, we propose a dialectical approach rooted in the Cultural-Historical Theory [31], the principles of dialectical thinking and the concept of metastable attunement as instructional design to enrich health professions education and to align IPE with IPCP more effectively. Through mediating activities that focus on dialectical inquiry in terms of contexts, processes of change and interrelations, students and healthcare professionals may become aware of the landscape of affordances while collaborating in a complex and dynamic healthcare practice.

We recommend that deans of faculty promote the dialectical approach to teaching and learning in health professions education and facilitate faculty development and educational research [4, 12]. In order to ensure a coherent continuum of learning, faculty staff must integrate the dialectical approach from the outset of the undergraduate curriculum. A gradual increase in the complexity of mediating activities may foster the growth in dialectical comments, reasoning, and praxis, and support metastable attunement to an unpredictable and uncertain environment in the complex and dynamic healthcare system. To that end, faculty development should enable healthcare professionals and educators to use the principles of dialectical inquiry as a pedagogy to optimally guide the long-term development of students in mastering dialectical praxis and more effectively align IPE to IPCP.

For optimal instructional design, it is important to examine the extent to which healthcare professionals and students employ dialectical thinking and demonstrate adaptive capacity within a complex and dynamic environment. The results may inform curriculum designers and educators to critically reconsider the structure and alignment of the learning continuum, apply more adaptive and interactive instructional design, and adjust faculty development accordingly. Other lines of research may concern the design and functioning of the mediating activities and the dialectical pedagogy in practice. Finally, follow-up research may be conducted to assess the impact of the dialectical approach on the performance of students, educators and healthcare professionals.

## Data Availability

No datasets were generated or analysed during the current study.

## Abbreviations

CanMEDS: Canadian Physician Competency Framework
CIHC: Canadian Interprofessional Health Collaborative
IPE: Interprofessional Education
IPCP: Interprofessional Collaborative Practice
WHO: World Health Organization
